# Transglutaminase 2 contributes to the blood clotting cascade

**DOI:** 10.1038/s41420-026-03388-1

**Published:** 2026-10-17

**Authors:** Mauro Piacentini, Nicola J. Mutch, Alessandro Mauriello, Manuel Scimeca, Mara Mancini, Valerio Da Ros, Francesco Barilla’, Yufang Shi, Eleonora Candi, Zhuo Wang, Martin Griffin, Gerry Melino

**Affiliations:** 1 Department of Experimental Medicine, University of Rome “Tor Vergata”, Rome, Italy; 2 Aberdeen Diabetes and Cardiovascular Centre, Institute of Medical Sciences, School of Medicine, Medical Sciences and Nutrition, University of Aberdeen, Aberdeen, UK; 3 Department of Biomedicine and Prevention, University Hospital of Rome “Tor Vergata”, Rome, Italy; 4 The Fourth Affiliated Hospital of Soochow University, Institutes for Translational Medicine, Suzhou Medical College, Soochow University, Suzhou, China; 5 Fondazione Luigi Maria Monti IDI-IRCCS, Rome, Italy; 6 Department of Biosciences, School of Health and Life Sciences, Aston Triangle, Aston University, Birmingham, UK

**Keywords:** Biochemistry, Pathogenesis

## Abstract

Cross-linking of fibrin and associated proteins via Nε-(γ-glutamyl)lysine bonds in clot formation is a well-established component of the hemodynamic mechanism, providing clot rigidity and reducing the rate of fibrinolysis. The latter effect occurs predominantly through the incorporation of fibrinolytic inhibitors. To date, this function has been attributed exclusively to Factor XIIIa, also known as plasma transglutaminase (TG), one of eight identified in mammals. In this report, we demonstrate that tissue transglutaminase (TG2), a ubiquitous enzyme abundantly present in erythrocytes, also contributed to clot stabilisation. We observed clear differences between whole thrombi isolated from TG2-deficient and wild-type mice. Similarly, human thrombi were more stable in the presence of TG2 than when TG2 activity is specifically inhibited, revealing a previously unrecognized role for this enzyme in haemostasis. Therefore, we propose TG2 as an additional factor involved in blood coagulation.

## Introduction

The crosslinking of fibrin is an essential part of the hemodynamic mechanism [1–4].To date fibrin stabilisation has been solely attributed to Factor XIIIa, a plasma transglutaminase (EC 2.3.2.13; TG) also found in platelets and monocytes [5].TGs are a family of enzymes that crosslink proteins via N^ε^(γ-glutamyl)lysine isopeptide bonds, leading to the formation of high molecular weight aggregates with increased rigidity and enhanced stability to proteolysis and chemical degradation [5–7].

The epidermis shows the presence of several TGs, such as TG1 [8] or TG5 [9], that participate in the formation of the cornified envelope [10] and therefore in tissue homeostasis [11, 12]. Deregulation of this process results in specific genetic diseases such as lamellar ichthyosis [8]. On the contrary, TG2 is the most ubiquitously expressed and functionally versatile isoform, showing a unique combination of enzymatic and non-enzymatic activities, as reviewed in [13–15]. In fact, serotonin can activate TG2 [16] in vascular smooth muscle cells [17], facilitating protein kinase A-induced phosphorylation of retinoblastoma protein [18] and can contribute to pulmonary inflammation and fibrosis in bleomycin-treated mice [19, 20]. Additional recent evidence, due to its crosslinking properties, has also implicated TG2 in cell death modalities [21]as well as Parkinson [22].

Abnormal blood clotting has been found in humans carrying genetic defects of Factor XIIIa [23]. However, this genetic defect is minor when compared to the phenotype of patients with defective Factor VIII or IX. A possible hypothesis indicates that there could be another TG that partially compensates for the defects of factor XIIIa. Indeed, TG2 is involved in wound healing [24] and it is highly abundant in erythrocytes and endothelial cells [5, 25]; therefore, it is present in clotted blood. Unlike Factor XIIIa, TG2 does not require proteolytic activation; it is found both in the intracellular and extracellular compartments and under normal physiological conditions its Ca^2+^ activated transamidating activity is kept silent inside the cell by its binding of GTP/GDP [26] or NO [27]. TG2-deficient mice [28] show minor phenotypes [29–32], one of which is slow wound healing. In this report we provide evidence for the involvement of TG2 in the blood clotting cascade.

## Results

**Fig. 1:**
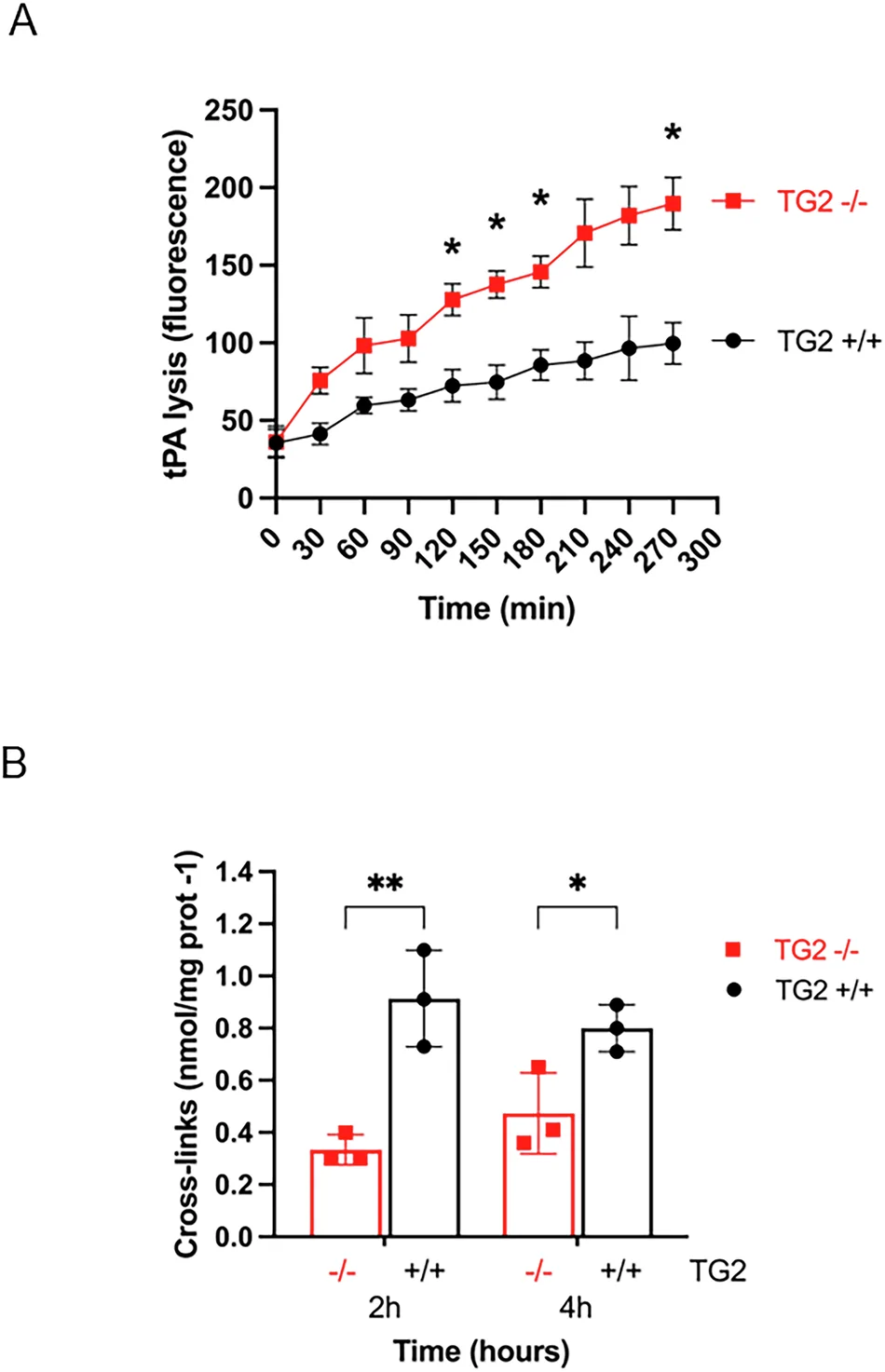
TG2 activity in mice. **A** The role of TG2 in thrombus stability. Thrombi were formed with blood from three WT (TG2^+/+^) and three TG2^–/–^ mice and lysed by tPA (250 ng/ml); the data shown are typical of three separate experiments, each with n.3 WT (TG2^+/+^) and n.3 TG2^–/–^ mice, with a significant difference in the rate of lysis at the indicated time points (*p < 0.05). Thrombi were bathed in 10 mM Tris pH 7.5, 0.01% Tween 20 containing 1 μg/ml tPA. Thrombi of mouse blood (drawn by cardiac puncture in 0.1 vol of 0.13 M trisodium citrate) were lysed by adding 250 ng/ml tPA. The *P* values were obtained using Anova and *p*-values < 0,05 were considered significant. **B** Cross-links in blood clots. Whole blood clots were formed with blood from TG2^–/–^ and WT (TG2^+/+^) mice. Clots were digested and analysed by HPLC. For analysis of the ε(γ-glutamyl)lysine isodipeptide, clots were first washed with PBS containing 5 mM EDTA and then with PBS alone. The bars represent the analyses of three WT (TG2^+/+^) and three TG2^–/–^ mice. The P-values were obtained using Student’s T-test. *p < 0.05, **p < 0.001.

The model thrombi [33] of TG2 null mice are more susceptible to haemolysis than thrombi prepared from wild-type mice (Fig. 1A). The rate of haemolysis was 2–3 fold greater (*p* < 0.001) than in wild-type animals and approximately 50% of the rate of haemolysis when the TG-specific inhibitor R283 was included in the clot mixture. Consistent with this change in haemolysis was the levels of N^ε^ (γ-glutamyl) lysine crosslink found in proteolytic digests of thrombi that was approximately 50% less (*p* = 0.05, 95%) in the TG2 KO mice, see Fig. 1B.

The presence of TG2 in the clot was confirmed in Supplementary Fig. 1 by staining of human model thrombi using a specific antibody for TG2. PCR analysis of leucocytes did not reveal the presence of TG2 mRNA and platelets contain very little TG2, suggesting that the erythrocyte is the major provider of TG2 for clot stabilisation.

**Fig. 2:**
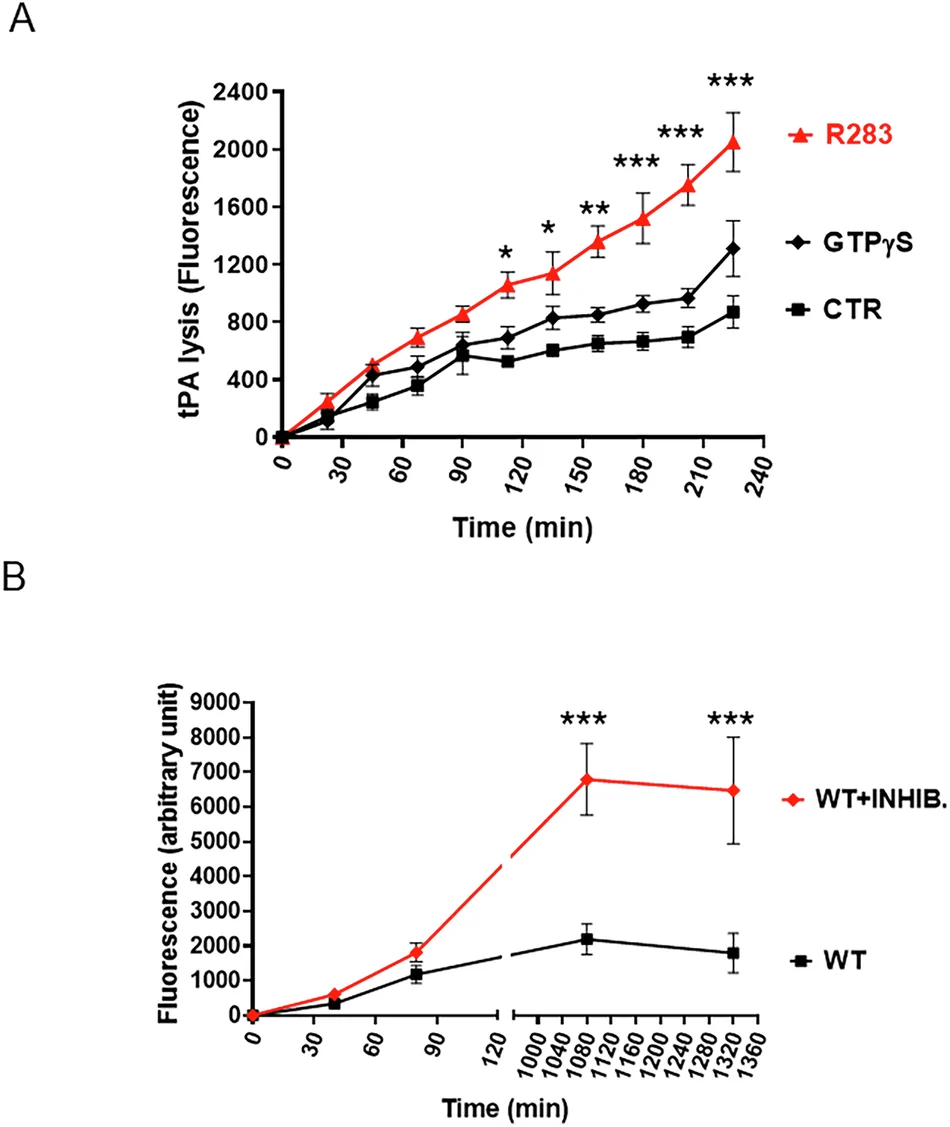
TG2 in human coagulation. **A** Inhibition of cross-linking in human thrombi. Thrombi were formed from normal human blood (CTR), ± 1 mM GTPγS, ± 1 mM R283 and lysis by tPA was monitored. Blood was collected from healthy, well-informed volunteers with their consent in 0.1 vol of 0.13 M trisodium citrate. GTPγS was incorporated into thrombi at a final concentration of 1 mM, a concentration shown to fully inhibit TG2. The TG active site-specific inhibitor 1,3-dimethyl-4,5 diphenyl-2 [2 (oxopropyl) thio] imidazolium inhibitor (R283) was added at the final concentration. **B** Thrombi were formed from normal mice blood (WT), ± 1 mM R283 (INHIB.) and lysis by tPA was monitored, as in (**A**) but at different times, comparing clots of control mice with or without the TG active site-specific inhibitor 1,3-dimethyl-4,5 diphenyl-2 [2 (oxopropyl) thio] imidazolium inhibitor (R283). The P-values were obtained using one-way ANOVA. *p < 0.05, **p < 0.001, ***p < 0,0001.

To further demonstrate the involvement of TG2 in thrombus stabilisation in humans, thrombi were prepared separately from blood of six normal human donors, incubated with the non-hydrolysable GTP analogue GTPγS. At 1 mM this nucleotide inhibits Ca2 + activation of TG2 but not that of Factor XIIIa, at the levels of Ca^2+^ used in the formation of the model thrombi [26]. Inclusion of GTPγS in the thrombi resulted in a 1.3 fold faster rate of lysis than control thrombi, as shown in Fig. 2A. Inhibition of all TG mediated crosslinking by the specific active site directed inhibitor R283 resulted in even faster lysis (1.7 fold, *p* < 0.005). This is also consistent with the crosslink levels and lysis data obtained from the mouse thrombi. Figure 2B shows that the treatment of mouse thrombi with the specific TG2 inhibitor results in a faster rate of lysis compared to the control. The data indicate that blocking TG2 results in less stable clots, suggesting functional cooperation between clotting factor XIIIa and TG2.

**Fig. 3:**
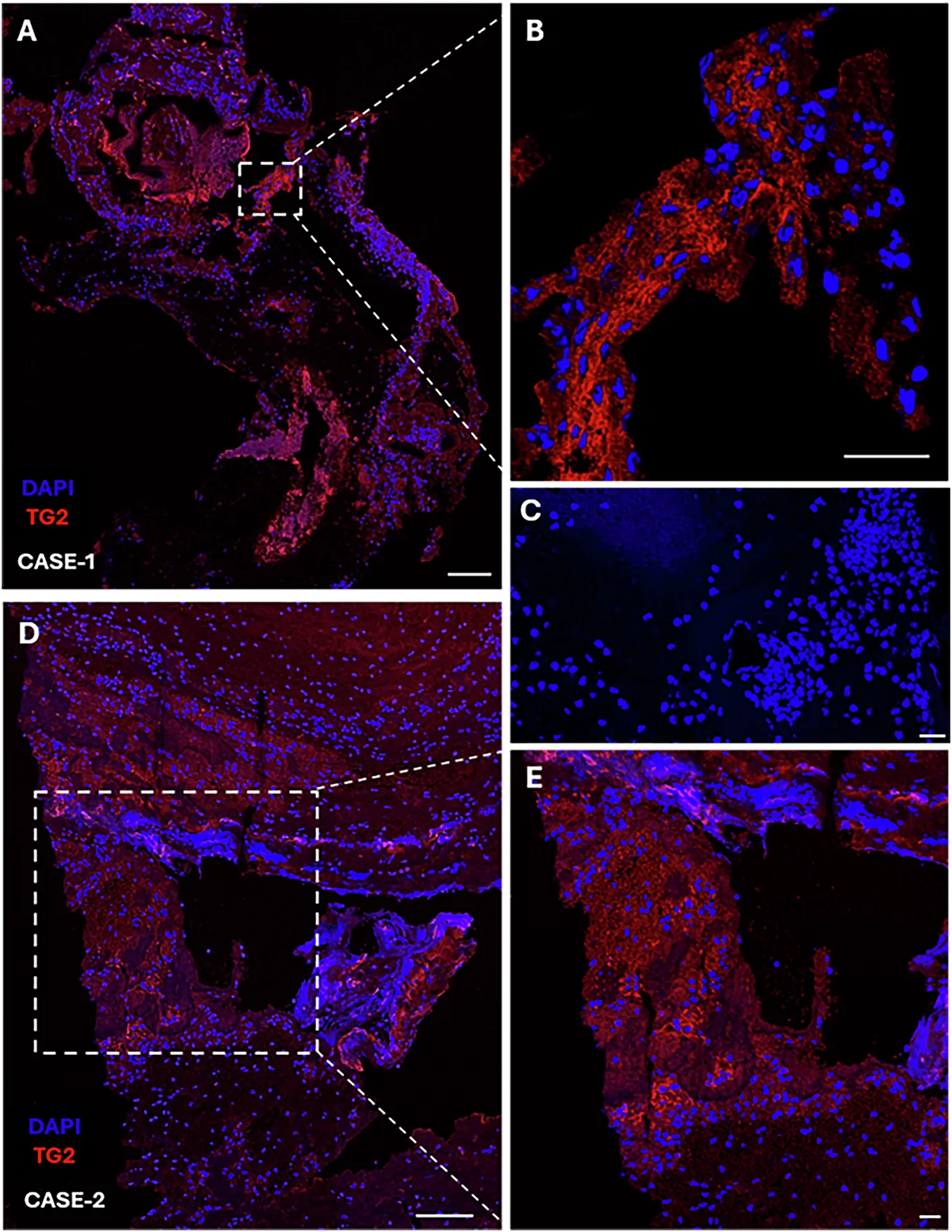
TG2 in human thrombi from brain stroke patients. **A, D** Large immunofluorescence staining showing TG2 expression (red) in case 1 and case 2 of the human thrombus. **B** High-magnification and super-resolution images of the indicated (white square) TG2- positive areas of the case 1 thrombus. **C** Negative control of the immunofluorescence staining of case1 obtained by incubating tissue slides only with Alexa Fluor 647 secondary antibody together with DAPI, without primary antibody. **D** High magnification of the indicated (white square) positive area of case 2. Scale bar is 100 µm (**A, D**), 50 µm (**B**) or 20 µm (**C, E**).

**Fig. 4:**
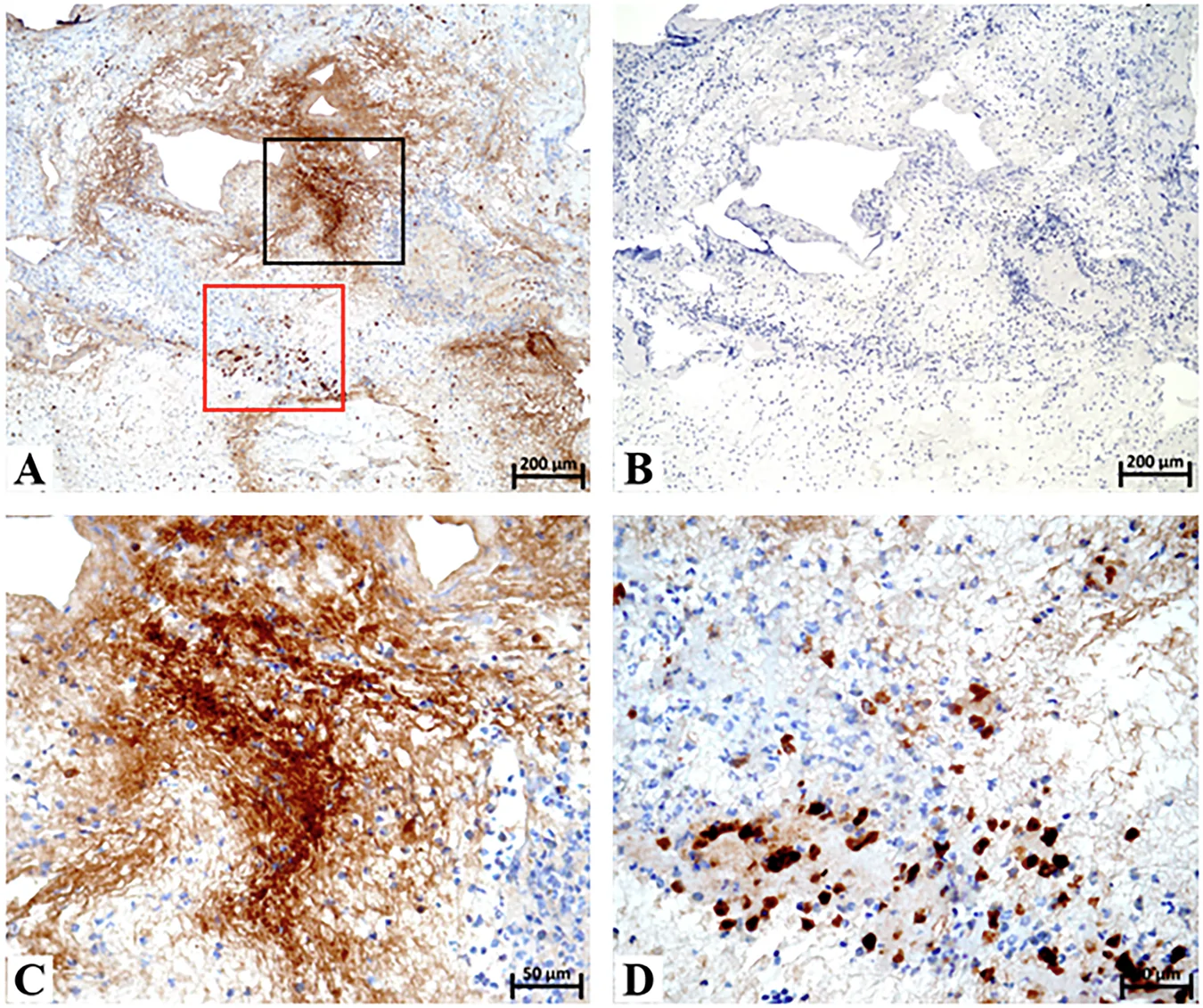
TG2 expression in human thrombi. **A** Representative immunohistochemical staining showing TG2 expression in a human thrombus; see Table 1 for clinical details. TG2 positivity is observed in both the extracellular compartment (black square) and blood cells (red square). **B** Negative control section processed without primary antibody incubation. **C** Higher magnification of the black square in panel A, showing intense TG2 signal within the fibrin-rich extracellular compartment. **D** Higher magnification of the red square in (**A**), highlighting several monocyte/macrophage cells within the thrombus. The scale bar is 200 μm (**A, B**) or 50 μm (**C, D**).

Having provided evidence in genetically modified mice, we then explored the presence of TG2 in 30 human thrombi collected during mechanical thrombectomy from patients with acute ischemic stroke. Table 1 shows the clinical details of the patients. Both immunofluorescence and immunohistochemical analyses confirmed the presence of TG2 in all human thrombi analysed (Table 1). Specifically, immunofluorescence revealed the location of TG2 within extracellular fibrin-rich regions of the clot (Fig. 3A–E). Immunohistochemistry also allowed the assessment of TG2 expression within the morphological context of the thrombus (Fig. 4A–D).

**Table 1:** Clinical and immunohistochemical data.

Case	Age	Gender	Thrombus site	Extracellular positivity (Score 0-3)	Blood cell positivity (Score 0-3)
1	82	M	anterior cerebral circulation	3	2
2	72	M	anterior cerebral circulation	3	1
3	57	M	anterior cerebral circulation	1	3
4	86	F	anterior cerebral circulation	3	1
5	93	F	anterior cerebral circulation	0	3
6	47	M	anterior cerebral circulation	1	0
7	75	M	anterior cerebral circulation	2	1
8	82	F	anterior cerebral circulation	3	2
9	85	F	anterior cerebral circulation	1	2
10	74	F	anterior cerebral circulation	3	1
11	67	F	anterior cerebral circulation	0	1
12	62	F	anterior cerebral circulation	1	1
13	71	F	anterior cerebral circulation	2	2
14	69	M	anterior cerebral circulation	2	0
15	91	F	anterior cerebral circulation	1	2
16	81	F	anterior cerebral circulation	1	3
17	89	F	anterior cerebral circulation	1	1
18	80	F	anterior cerebral circulation	1	0
19	56	M	anterior cerebral circulation	3	1
20	80	F	anterior cerebral circulation	1	1
21	79	F	anterior cerebral circulation	1	2
22	81	F	anterior cerebral circulation	0	2
23	76	F	anterior cerebral circulation	3	0
24	86	F	anterior cerebral circulation	2	0
25	85	M	anterior cerebral circulation	1	1
26	76	M	anterior cerebral circulation	2	1
27	88	M	anterior cerebral circulation	1	1
28	71	M	Posterior cerebral circulation	2	2
29	93	M	anterior cerebral circulation	1	1
30	50	M	anterior cerebral circulation	1	0

TG2 staining showed marked heterogeneity between samples. All thrombi exhibited positivity for TG2 in the extracellular compartment (fibrin-rich regions) and/or within blood cells. Regarding the extracellular compartment (Fig. 4A, C), extracellular TG2 expression was detected in 27 of the 30 thrombi analysed. Specifically, 7 thrombi showed strong positivity (score 3), 6 moderate positivity (score 2) and 14 weak positivity (score 1), while only 3 thrombi did not show detectable extracellular TG2 staining (score 0). In particular, extracellular TG2 positivity was predominantly observed in areas characterised by extensive haemolysis.

Cellular TG2 expression was also highly heterogeneous, although TG2-positive cells were predominantly represented by cells of the monocyte/macrophage lineage (Fig. 3D). Specifically, 3 of the 30 thrombi showed strong cellular positivity (score 3), 8 moderate positivity (score 2) and 12 weak positivity (score 1). The remaining 7 thrombi were negative for cellular TG2 staining (score 0). Immunofluorescence and immunohistochemical analysis demonstrate that TG2 is detectable in human ischemic stroke thrombi, although with substantial interpatient variability in both extracellular and cellular distribution. Differences in TG2 abundance or localisation might reflect distinct thrombus compositions, stages of thrombus organisation, cellular contributions, etiological mechanisms, or responses to vascular injury. Therefore, TG2 expression could potentially contribute to the molecular characterisation of thrombi and, following validation, could complement established clinical, radiological and histopathological parameters.

**Fig. 5:**
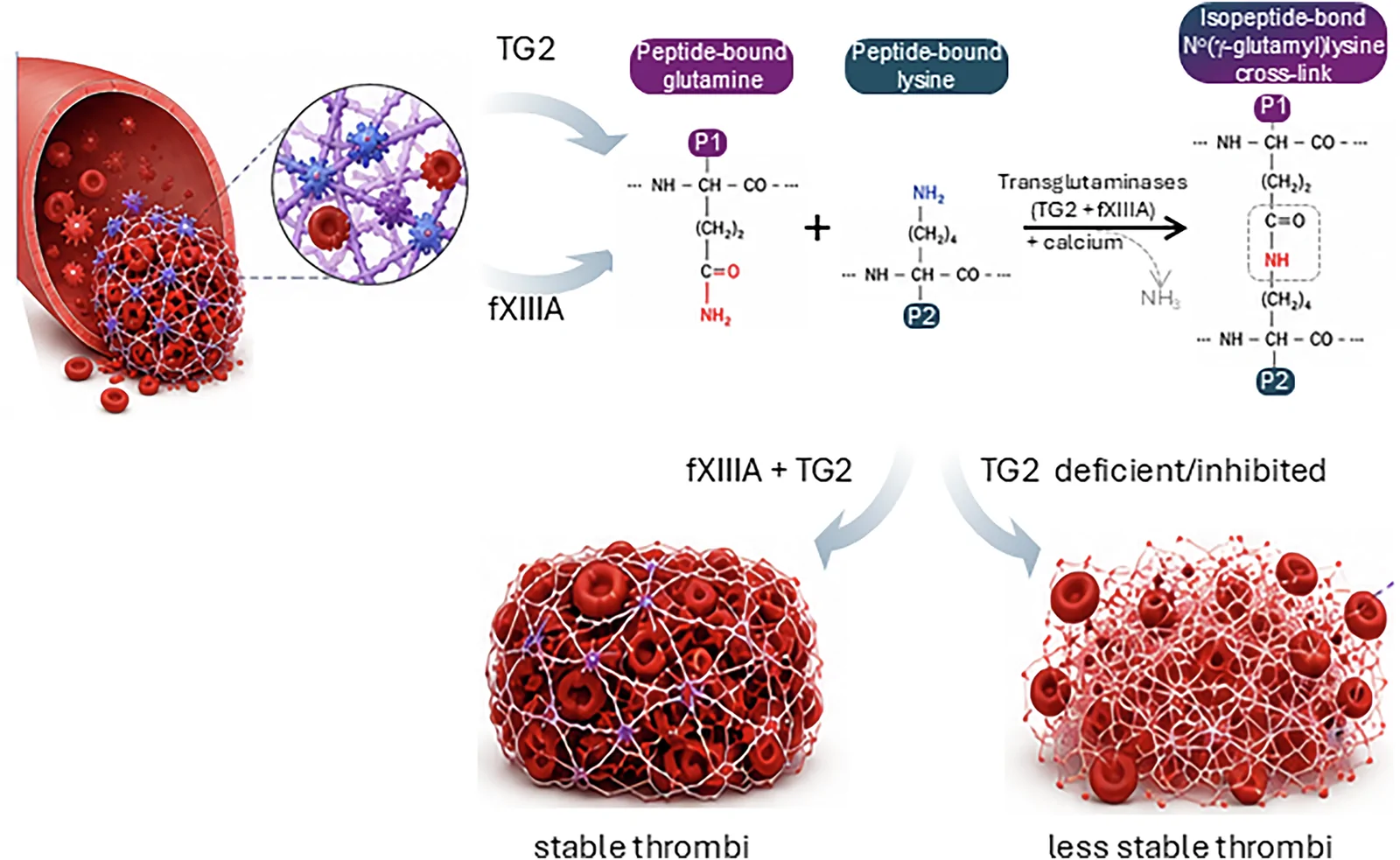
TG2 contributes to haemostasis. During the formation of the clot, fibrin and other proteins become cross-linked by factor XIIIA, providing rigidity and stability to the clot, including tight regulation of fibrinolysis. This function is provided by clotting factor XIIIA, which belongs to the family of transglutaminases. In addition to factor XIIIA, TG2, abundant in erythrocytes, catalysis e- (g-glutamil) lysine crosslinks in fibrin and associated proteins. Evidence from TG2-deficient mice and human thrombi treated with a specific TG2 inhibitor demonstrates that loss or inhibition of TG2 results in less stable clots. Therefore, we propose that TG2 is able to contribute to the cross-linking of fibrin and associated proteins, promoting clot stability and modulating fibrinolysis. These findings support the involvement of TG2 as an additional supporting factor in the blood coagulation cascade.

These data provide clinical relevance for TG2 in human clots, as depicted in Fig. 5.

## Discussion

The results presented here clearly demonstrate that TG2 participates in the cross-linking and stabilisation of the fibrin network in the initial event of forming the haemostatic plug. This could explain the mild phenotype demonstrated by patients deficient in factor XIIIa: the presence of TG2 in the blood cascade mechanism would at least partially compensate for this deficiency. To date, the role of TG2 in erythrocytes has been elusive and the enzyme was never found to be activated in red cells, nor was any in vivo substrate identified. Mature erythrocytes are enucleated cells with limited protein synthesis and are commonly considered to have a relatively restricted enzymatic repertoire. Furthermore, under resting intracellular conditions, TG2 transamidase activity is generally suppressed by low cytosolic calcium concentrations and the presence of guanine nucleotides. Activation may occur only in specific pathological or experimental contexts associated with calcium influx, oxidative stress, membrane damage, energy depletion, cell ageing, or loss of membrane asymmetry. Such conditions can develop during thrombus formation but can be transient, spatially restricted, or confined to a subpopulation of erythrocytes. Consequently, circulating erythrocytes under resting conditions may fail to detect active TG2. Our results are consistent with the possibility that in erythrocytes, TG2 becomes functionally relevant under the perturbed conditions that occur during clot formation rather than in resting circulating cells. Moreover, it indicates the importance of this step in the stabilisation of the clot and wound healing as a whole, since mammals have evolved not one but two separate enzymes that are capable of clot stabilisation and which are activated by different mechanisms, one by proteolysis by thrombin, the other by Ca^2+^ after its release after lysis of red cell lysis in the clot. This phenomenon is reminiscent of the process that occurs during epidermal terminal differentiation, where several transglutaminases contribute to the formation of the cornified envelope, which provides protection to the organism [10]. From an evolutionary standpoint, the conservation and diversification of multiple members of the transglutaminase family indicate their critical contribution to fundamental biological processes. Their distinct but overlapping functions support key protective mechanisms, including haemostasis, barrier integrity, tissue remodelling and stress adaptation [34–38], highlighting the evolutionary advantage of maintaining a repertoire of specialised transglutaminases to safeguard organismal homeostasis.

Important physiological differences in the cross-linking specificity of these enzymes have come from studies on purified fibrinogen [39]. Once activated by thrombin, Factor XIIIa rapidly cross-links γ-chains first into γ-γ dimers and then polymers, but it acts slowly in the formation of α-polymers and intermolecular cross-links between α and γ chains. On the contrary, TG2 acts rapidly in the formation of α-polymers and the formation of intermolecular cross-links between α and γ chains. The TG2 cross-linking bridges the Aα (408-421) chain segment to γ (392-406), thus binding the flexible αC domain closer to the fibrinogen backbone. These differences in specificity are likely to be of crucial importance to clot rigidity in the initial formation of the haemostatic plug and in its subsequent stability during the formation of the granulation tissue during wound healing. Studies indicate that clot rigidity is strongly correlated with the formation of α_n-_polymers_,_ αpγq—hybrid crosslinks and γ multimers [40, 41]. In contrast, the initial formation of γ-γ dimers by factor XIIIa contributes very little to clot rigidity. Both TGs are able to cross-link inhibitors of fibrinolysis to fibrinogen and fibrin, but with slightly different preferences. Both enzymes cross-link PA1-2 and TAFI to fibrinogen/fibrin, but Factor XIIIa is reported to be more efficient in cross-linking α2-antiplasmin.

As expected, no role for TG2 was observed when plasma was clotted in the absence of blood cells, as is commonly the case. These white clots were lysed at comparable rates whether prepared from TG2-deficient or wild-type mice. This is consistent with the current belief that Factor XIIIa is the major fibrin stabilising factor; a view which arose from studies on plasma alone. Suggestive or correlative evidence linking TG2 to cardiovascular events has previously been reported. In fact, TG2 appears to protect cardiomyocytes against ischemia/reperfusion injury by regulating ATP synthesis [42]. Consequently, anti-TG2 antibodies have been detected in acute coronary syndrome [43]. Although the original interpretation suggested the involvement of TG2 in platelet aggregates as a response to lipid plaque, our new data suggest a causal role of TG2, triggered by the release of few lysed erythrocytes, to expand the red clot, thus forming an overt thrombus. These data seem to involve TG2 in the larger field of cardiovascular diseases [44–46].

Although our work was in its final stages of refined preparation, two very recent reports have described a role for TG2 in thrombus formation [47, 48]. Boateng and collaborators [47] have shown that TG2 is capable of driving fibrin β-chain cross-linking. In their hands, fibrinogen is an efficient substrate for TG2, allowing the formation of β−α cross-links, at glance with the cross-links by Factor XIII (that is α−α, β−β). The authors describe these TG2-directed β-chain cross-links in trauma patients and not in clots from healthy control individuals. Chahdi et al. [48] also provide evidence that TG2 contributes to the generation of thrombin and clot formation, suggesting a role for TG2 in fibrin structure and spatial distribution within blood clots. Here, also, TG2 promotes faster thrombin generation and clot formation. However, its interpretation at the molecular level involves mechanical forces exerted by platelets that allow TG2 to be released by erythrocytes, which then cross-links fibrinogen [1, 49]. Together with our findings, these studies suggest that TG2 may influence thrombus formation through multiple context-dependent mechanisms. The detection of TG2 in thrombi recovered from patients with acute ischemic stroke further supports the potential relevance of these mechanisms in human arterial thrombosis. From a translational perspective, TG2 could represent a candidate biomarker of thrombus composition or a potential pharmacological target in conditions characterised by aberrant clot formation.

TG2 has already been involved in injured and remodelling cardiac tissues, without evoking any clotting activity, where it seems to modulate cellular phenotype, while contributing to matrix crosslinking [11, 50–52]. Similarly, TG2 seems to exert angiogenic properties by its transamidation function [25]. However, the most direct evidence of interaction between the function of TG2 and clotting factor XIII comes from double knockout mice for both TG2 and FXIII-A, showing a synergistic action in cross-linking activities [53]. In addition, TG2 moderates the expansion of mouse abdominal aortic aneurysms [54] and TG2 limits extravasation and the resultant myocardial fibrosis associated with factor XIII-A deficiency [55], further demonstrating the Factor XIIIa or TG2 interaction. FXIII A subunit (FXIII-A) knockout mice show that platelet aggregation induced by adenosine diphosphate or collagen was normal, while clot retraction (CR) was lost in platelet-rich plasma (PRP); in contrast, there was no CR impairment in the PRP of TG2-knockout mice compared to wild-type mice, indicating that the enzymatic activity of FXIIIa could potentially be synergistic [56].

During thrombosis, activated platelets, leukocytes and coagulation factors generate a highly pro-oxidant and pro-inflammatory microenvironment characterised by reactive oxygen species (ROS), cytokine release, calcium influx and fibrin accumulation. These stimuli strongly induce TG2 expression and activation in endothelial cells [57]. TG2 activation contributes to endothelial dysfunction and cell death [57, 58]: under severe ischemic or inflammatory conditions, excessive TG2 activity can favour apoptosis or necrotic-like cell death, exacerbating vascular occlusion and thrombo-inflammatory injury. These observations suggest that TG2 acts as a stress-responsive regulator at the interface between coagulation, inflammation and endothelial survival during thrombotic disease [59].

By examining a complete model including whole blood, together with some additional very recent work [47, 48], here, we demonstrate a definitive functional role for TG2 in the blood cascade mechanism, Fig. 5, in keeping with the other roles for this enzyme in wound healing. As a consequence, TG2 could be seen as ‘Factor XIV’, supporting Factor XIII in the blood cascade mechanism.

## Materials and methods

### Mice

As previously described [28, 60], hemizygous 14E06 Ig KI mice were generated on a C57Bl/6 background by crossing VDJH14E06 and VJκ14E06 single KI mice. TG2–/– mice were generated as described by De Laurenzi & Melino; VDJH14E06 and VJκ14E06 single KI mice were crossed to Tgm2 − /− mice [28]. See also the protocols in [12, 61]. Mice were reared in the animal house at the Department of Experimental Medicine (University of Rome Tor Vergata). The animals were housed individually in an environmentally controlled room (18–21 °C, 40–70% relative humidity) with ad libitum access to water and food and kept in a 12-h dark/12-h light cycle. All animal procedures were approved by the Ethics Commission of the University of Rome ‘Tor Vergata’ and performed according to the principles established by the National Institutes of Health and by the Italian Ministry of Health.

### Thrombi

Blood samples were collected from normal or knockout mice with their consent into 0.1 vol of 0.13 m trisodium citrate, as described [33, 62]. Plasma that was essentially platelet-free was prepared by centrifugation at 1860 × *g* for 30 min at 4 °C. The normal pool plasma, used in the clot lysis experiments, was from 3 wild-type or 3 knockout mice [33]. For the Clot lysis assay, as described [33], pooled plasma in the presence and absence of CPI and the plasminogen activators tPA (Genentech, San Francisco, CA, USA), uPA (NIBSC) or scuPA (Abbott, Maidenhead, Berks, UK) or plasmin (NIBSC, Potter’s Bar, UK) was prepared in a final volume of 200 µL 10 mm Tris pH 7.5, 0.01% Tween 20 (Tris–Tween buffer). Two 80 µL aliquots from each tube were added to microtiter wells, each containing 20 µL CaCl2 to give the final concentrations in the wells as follows: 30% plasma, 10.6 mm CaCl2; 180 pm tPA, 1 nm uPA, 8 nm scuPA or 1.25 U ml-1 plasmin; ± 25 µg mL−1 CPI. The plate was incubated at 37 ° C and read continuously at 405 nm [33, 62].

### Cross-linking experiments

Cross-linking experiments were performed as previously described [6, 63]. See also the protocols in [64]. Briefly, enzymatic activity was measured by incorporation of [3H] putrescine (Amersham Corp., specific activity 26 Ci/mmol) into succinylated casein and the amount of TG2 incorporated 0.45 pmol/min in casein was used. Cross-linked products were separated on 4–12% polyacrylamide gels and analysed by autoradiography [65, 66]. The Nε-(γ-glutamyl)lysine crosslink was analysed as detailed in the legend to Fig. 1: Crosslinks in blood clots. Whole blood clots were formed with blood from TG2^–/–^ and WT (TG2^+/+^) mice. Clots were digested and analysed by HPLC. For analysis of the Nε−(γ-glutamyl)lysine isodipeptide bonds, clots were first washed with PBS containing 5 mM EDTA and then with PBS alone. The clots were then lyophilised and resuspended in 0.1 M ammonium hydrogen carbonate pH 8 buffer and then proteolytically digested) and analysed using cation exchange chromatography as described by Griffin and Wilson [66].

### Human cohort

Thirty thrombi were collected during mechanical thrombectomy from patients with acute ischemic stroke caused by large vessel occlusion of the intracranial anterior circulation; see Table 1. As previously described [62], thrombi were fixed in 10% neutral-buffered formalin and embedded in paraffin. Serial 4-μm-thick sections were prepared for haematoxylin and eosin staining, immunofluorescence and immunohistochemical analyses.

For immunofluorescence analysis, tissue deparaffinized sections were rehydrated in graded alcohol and immersed for 1 h in 70% ethanol supplemented with 0,25% NH_3_ to remove tissue autofluorescence [67]. Slide rehydration was resumed by immersion in 50% ethanol for 10 min, after which the sections were washed with distilled H_2_O and underwent heat-induced antigen retrieval in EDTA-citrate buffer (pH 6.8) using a pressure cooker at 120 °C for 15 min. The sections were then blocked for 1 h at room temperature with 5% goat serum and then incubated for 1 h with mouse monoclonal anti-TG2 antibody (1:500; clone CUB 7402; Invitrogen). Immunoreactivity was detected using Alexa Fluor 647 as an anti-mouse secondary antibody (Thermo Fisher) and DAPI (Sigma-Aldrich) was used to counterstain the cells' nuclei. The fluorescence was detected and acquired using a Leica Stellaris 5 and images were analyzed with LASX Leica software.

Immunohistochemical staining was performed using the automated Leica Bond III staining platform (Leica Biosystems). The same primary anti-TG2 antibody used for immunofluorescence (1:1000; clone CUB 7402; Invitrogen) was applied under identical conditions. Immunoreactivity was visualised using a horseradish peroxidase (HRP)-based detection system with 3,3′-diaminobenzidine (DAB) as the chromogen. TG2 immunoreactivity was assessed separately in the fibrin-rich extracellular component and in the cellular component of each thrombus using a semiquantitative pathological scoring system. For the extracellular compartment, staining was scored as follows: score 0, no TG2-positive fibrin-rich areas; score 1, TG2 positivity involving 1–10% of the fibrin-rich area; score 2, TG2 positivity involving 11–20% of the fibrin-rich area; and score 3, TG2 positivity involving >20% of the fibrin-rich area. For the cellular compartment, the staining was scored as follows: score 0, absence of TG2-positive cells; score 1, TG2-positive cells accounting for 1–10% of the cellular population; score 2, TG2-positive cells accounting for 11–20% of the cellular population; and score 3, TG2-positive cells accounting for >20% of the cellular population.

### Statistical analysis

Statistical analysis was performed using Student’s *t* test and one-way ANOVA, as also specified in the figure legends. All analyses were performed using Prism v.8.0 (GraphPad Software, La Jolla, CA, USA). Values were expressed as mean ± S.D. and statistical significance was assumed at a *p* value of 0.05 or less (*=*p* < 0.05; **=*p* < 0.01; ***=*p* < 0.001; ****=*p* < 0.0001).

## Supplementary information

Supplementary figure 1

## Data Availability

The data supporting the findings of this study are available from the corresponding author upon reasonable request.
